# Second thoughts on the final rule: An analysis of baseline participant characteristics reports on ClinicalTrials.gov

**DOI:** 10.1371/journal.pone.0185886

**Published:** 2017-11-06

**Authors:** Amos Cahan, Vibha Anand

**Affiliations:** IBM T.J Watson Research Center, Yorktown Height, NY, United States of America; University of Illinois-Chicago, UNITED STATES

## Abstract

**Background:**

ClinicalTrials.gov is valuable for aggregate-level analysis of trials. The recently published final rule aims to improve reporting of trial results. We aimed to assess variability in ClinicalTirals.gov records reporting participants’ baseline measures.

**Methods and findings:**

The September 2015 edition of the database for Aggregate Analysis of ClinicalTrials.gov (AACT), was used in this study. To date, AACT contains 186,941 trials of which 16,660 trials reporting baseline (participant) measures were analyzed. We also analyzed a subset of 13,818 Highly Likely Applicable Clinical Trials (HLACT), for which reporting of results is likely mandatory and compared a random sample of 30 trial records to their journal articles. We report counts for each mandatory baseline measure and variability reporting in their formats. The AACT dataset contains 8,161 baseline measures with 1206 unique measurement units. However, of these 6,940 (85%) variables appear only once in the dataset. Age and Gender are reported using many different formats (178 and 49 respectively). “Age” as the variable name is reported in 60 different formats. HLACT subset reports measures using 3,931 variables. The most frequent Age format (i.e. mean (years) ± sd) is found in only 45% of trials. Overall only 4 baseline measures (Region of Enrollment, Age, Number of Participants, and Gender) are reported by > 10% of trials. Discrepancies are found in both the types and formats of ClinicalTrials.gov records and their corresponding journal articles. On average, journal articles include twice the number of baseline measures (13.6±7.1 (sd) vs. 6.6±7.6) when compared to the ClinicalTrials.gov records that report any results.

**Conclusions:**

We found marked variability in baseline measures reporting. This is not addressed by the final rule. To support secondary use of ClinicalTrials.gov, a uniform format for baseline measures reporting is warranted.

## Introduction

ClinicalTrials.gov is an invaluable resource for researchers, clinicians and patients. Across-trial secondary analysis is made possible using the Database for Aggregate Analysis of ClinicalTrials.gov (AACT). [1] In theory, this resource allows for pooling of trial results around a specific disease or intervention, or studying trends in research over time. However, any such pooling of trial results requires not only that results are reported but also that the data types reported and their format are aligned.

The ClinicalTrials.gov registry holds over 224,000 studies, with 23,000 of them reporting summary results. [2] Made public in September 2016,[3] the final rule is a new regulation developed by the Health and Human Services (HHS) attempting to clarify the requirements for reporting of summary results in ClinicalTrials.gov registry in order to increase reporting. The National Institutes of Health (NIH) simultaneously issued a complementary final policy covering trials funded by the NIH. Under this policy, results are to be reported for all NIH-funded clinical trials, including those exempt from the Food and Drug Administration Amendments Act of 2007 (FDAAA requirements). [4]

Originally, mandatory registration of Interventional studies of drugs, biologics, or devices was covered under the FDAAA. Section 801of the FDAAA mandates that all covered studies report results within 1 year of completion. [5] At the minimum, FDAAA requires reporting of participants’ age and gender using a semi-structured format. Customized “Study-Specific Measures” may be also reported at the discretion of the trial sponsor or principal investigator.[6]

The final rule of 2016 [3] expands these reporting requirements. Reporting of either race or ethnicity is required, as is reporting of baseline measures analyzed with regard to the primary outcome measure of the clinical trial. Under the final rule, all trials completed after January 17, 2017 are subject to these mandatory reporting of results.

In this work, we evaluate the degree to which baseline measures reported for trials on ClinicalTrials.gov can be pooled for secondary analysis in their original format—first by analyzing counts and formats of mandatory baseline participant measures and second by comparing these to those reported in published journal articles (using a random sample) in literature.

## Methods

### Requirements for results reporting in the final rule

Mandatory baseline measures: According to the final rule, mandatory baseline measures to report are age, sex/gender, ethnicity and region of enrollment. In addition, any number of study-specific baseline measures can be reported [7]. Age reporting can be continuous, categorical or customized. Sex/gender can be reported as “sex, male, female” and/or “gender, customized”. Race reporting options include standard NIH and U.S. Office of Management and Budget Classification (NIH/OMB) format or a customized race/ethnicity format.

For each baseline measure, the following three elements are to be included: “(a) Name and description of the measure, including any categories used; (b) Measure Type (one of: “Standard deviation,” “inter-quartile range,” “full range,” and “not applicable”) and Measure of Dispersion (taken from: “count of participants,” “count of units,” “number,” “mean,” “median,” “least squares mean,” “geometric mean,” and “geometric least squares mean.”); and (c) Unit of Measure. Furthermore, data should be reported by study arm or comparison group and overall study population.

### Dataset

The September 2015 version of AACT [1] was used for the analyses in this study. This database includes all trial records found on ClinicalTrials.gov. Please note, studies included in this database were not subject to the final rule. We analyzed two sets of trials: (a) studies reporting results from the entire ClinicalTrials.gov registry; and (b) a subset of studies, highly likely applicable clinical trials (HLACTs), [8] for which reporting of results is likely to have been mandatory. The latter subset was derived by applying an algorithm to the subset in (a). This algorithm was proposed by Anderson et al [9] but for our analyses we removed a constraint to include trials completed after 2012. Analysis of both datasets (a) and (b) included aggregation of various reporting formats for the most frequent data types. Also included in this analysis are counts of unique baseline measures and their formats—that are reported by one or more trials. For brevity, the primary author (AC) manually reviewed the list of baseline measures and grouped together those with same semantics but different names (e.g. “kilogram per square meter“, and “kg/m^2“).

### Assessing agreement between ClinicalTrials.gov and published article

We used HLACT studies to assess agreement between ClinicalTrials.gov and published journal articles. A random sample of completed HLACTs was generated to assess the degree of agreement (i.e. compare if baseline measures reported in ClinicalTrials.gov is in concordance with corresponding journal articles). The first 30 trials in the sample associated with an open access journal article were used for comparison. A PubMed (www.pubmed.com) search using the ClinicalTrials.gov identifier (NCT number) was used to identify the corresponding journal articles. We manually compared: (a) Age and Gender reporting formats, and (b) the total number of baseline measures reported in each trial. The latter was chosen as a gross measure for discrepancy in reporting of results between the two datasets.

## Results

### Clinical trials reporting baseline characteristics

**Fig 1:**
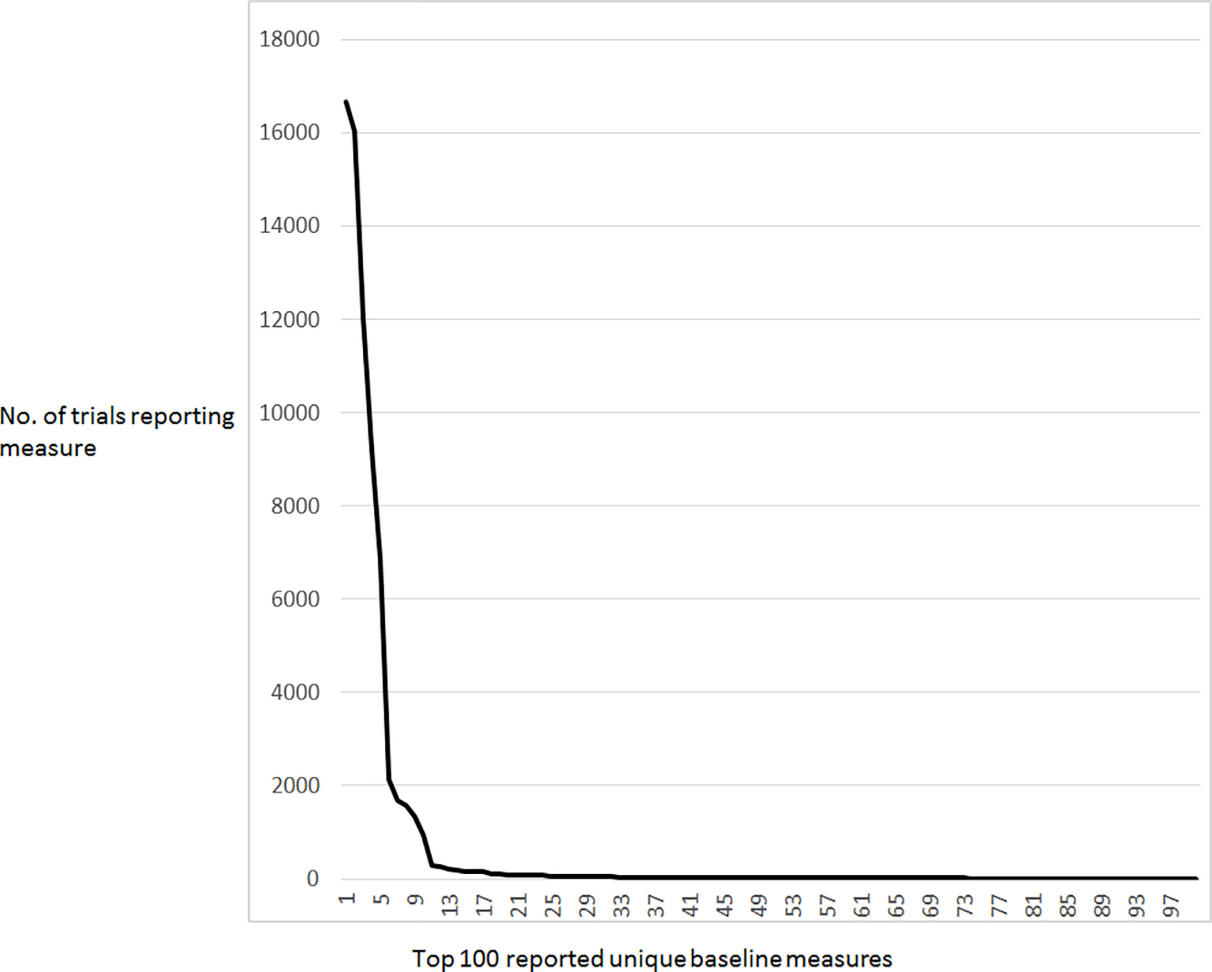
Frequency of reporting the top 100 unique baseline measures among trials with results listed on ClinicalTrials.gov.

There are 186,941 unique trials in the AACT version of database used for this study. Of these, 16,660 trials (8.9%) report baseline participant measures and 13,818 are HLACTs (S1 Table). There are 8,161 unique variables used in baseline measures reports and 1,206 unique “units of measurement” for baseline measures in the data dictionary. Fig 1 shows the frequency distribution for the top 100 reported unique baseline measures in the AACT database. Of note, 6,940 (85%) baseline measures are reported only once in the entire registry.

Table 1 lists the most common baseline measures aggregated on “dispersion” and “units of measurement”. Of note, since a trial may report a baseline measure in more than one format, the total count of reports in the Table could be higher than the number of reporting trials. We found “Number of Participants” reported 100% of the times in trials reporting results, followed by “Gender” and “Age” as most frequently reported measures (98% and 93% respectively).

**Table 1:** Frequency of top aggregated baseline measures reported in >1 study on ClinicalTrials.gov (N = 16,660).

Baseline measure type (aggregated)	No.(%) of trials reporting
Number of Participants	16660 (100%)
Gender	16362 (98%)
Age	15471 (93%)
Region of Enrollment	9267 (56%)
Race/Ethnicity, Customized	2193 (13%)
Age, Customized	1938 (12%)
Race (NIH/OMB)	1372 (8%)
Ethnicity (NIH/OMB)	961 (6%)
Weight	667 (4%)
Body mass index	499 (3%)
Height	391 (2%)
Gender, Customized	302 (2%)
Performance status	273 (2%)
Hemoglobin A1c	221 (1%)
Smoking Status	173 (1%)
Fasting Plasma Glucose	116 (1%)
Duration of diabetes	101 (1%)
Systolic Blood Pressure	82 (<1%)
Diastolic Blood Pressure	77 (<1%)

Table 2 shows the most frequent baseline measures in records of trials reporting results. Here, 9 (47%) of the top 19 measures listed are formats of mandatory measures. However, many different formats here are used for reporting of Age and Gender. In fact 178 for representing Age and 49 for reporting Gender respectively (S2 Table). Furthermore, “Age” alone as the exact variable name is reported in 60 different formats (e.g. title: “Age”; units of Measurement:” weeks”; measure type: “median”; dispersion: “full range”).

**Table 2:** The most frequently reported baseline measures of participants in studies with results listed in ClinicalTrials.gov (N = 16,660) by units and dispersion.

Baseline measure	Units	Dispersion	Number of studies reporting measure (%)
Number of Participants	participants		16660 (100)
Gender	participants		16012 (96)
Age	Years	SD	11996 (72)
Region of Enrollment	participants		9205 (55)
Age	participants		6814 (41)
Race/Ethnicity, Customized	Participants		2002 (12)
Age, Customized	participants		1671 (10)
Age	Years	Range	1589 (9)
Race (NIH/OMB)	participants		1344 (8)
Ethnicity (NIH/OMB)	participants		948 (6)
Gender, Customized	participants		285 (2)
Weight	Kg	SD	254 (1)
Gender	Subjects		194 (1)
Age	Months	SD	178 (1)
Age	Years	IQR	153 (1)
Height	Cm	SD	152 (1)
Body Mass Index (BMI)	kg/m^2	SD	151 (1)
Body Mass Index	kg/m^2	SD	111 (1)
Weight	Kilograms	SD	100 (1)
Age, Customized	Years	SD	86 (<1)

NIH/OMB, U.S. National Institutes of Health and U.S. Office of Management and Budget Classification Categories; SD, standard deviation; IQR, inter-quartile range

### Highly likely applicable clinical trials (HLACTs)

Highly Likely Applicable Clinical Trials use 3,931 variables in baseline measures reports. The top baseline measures reported are listed in Table 3. There are 6 different formats for reporting Age, 4 for Race/ Ethnicity, and 3 for Gender. S3 Table lists the most frequently reported baseline measures for HLACTs (aggregated by “units of measurement” and “dispersion”). Interestingly, diversity in baseline reporting is found even in this highly selective set of studies. Although Age (after aggregation) is reported in majority of studies (62% of HLACTs), Number of Participants and Gender are less frequently (45%) reported overall. Only 4 baseline measures (Region of Enrollment, Age, Number of Participants, and Gender) are reported by at least 10% of HLACTs.

**Table 3:** The most frequently reported baseline measures of participants in highly likely applicable clinical trials (HLACT)(N = 13,808) by units and dispersion.

Baseline measure	Units	Dispersion	Number of studies reporting measure (%)
Number of Participants	Participants		6260 (45)
Gender	Participants		6086 (44)
Age	Years	SD	4324 (45)
Region of Enrollment	Participants		3865 (31)
Age	Participants		2743 (20)
Age	Years	Range	746 (5)
Race/Ethnicity, Customized	Participants		639 (5)
Age, Customized	Participants		617 (4)
Race (NIH/OMB)	Participants		593 (4)
Ethnicity (NIH/OMB)	Participants		424 (3)
Weight	Kg	SD	133
Gender	Subjects		93 (1)
Age	Years	IQR	61 (<1)
Body Mass Index	kg/m^2	SD	55 (<1)
Height	Cm	SD	51 (<1)
Gender, Customized	Participants		50 (<1)
Age	Months	SD	46 (<1)
Race/Ethnicity	Participants		45(<1)
Age, Customized	Years	Range	44 (<1)
Eastern Cooperative Oncology Group (ECOG) Performance Status	Participants		40 (<1)

NIH/OMB, U.S. National Institutes of Health and U.S. Office of Management and Budget Classification Categories; SD, standard deviation; IQR, inter-quartile range

### Comparison with published articles

Manual comparison of ClinicalTrials.gov records and corresponding journal articles (S4 Table) reveals discrepancies in both the types and formats of reported measures. Whereas Age is reported using a consistent format in the majority of publications and their corresponding trials (23 of 30 or 77%), Gender is not (5 of 30 or 17%). This may be due to Gender mismatch in reporting, for example, use of “number” of “male” and “female” participants in ClinicalTrials.gov and “% male” or “% female” in the corresponding paper. Whereas differences in the format of baseline measures reports may have no clinical significance, they limit automatic pooling of data from across trials. Overall, journal articles include, on average, about twice as many additional characteristics when compared to ClinicalTrials.gov reports (mean±sd: 13.6±7.1 vs. 6.6±7.6, respectively).

## Discussion

In this work, we analyzed studies registered on ClinicalTrials.gov to assess reporting patterns of participant’s baseline measures. Overall, our findings suggest inconsistent reporting of these measures. As in other work[8–10] we found overall reporting rates of less than 10%. Even among HLACTs, which are trials highly likely to report results, less than half report Gender, a mandatory baseline measure. Among trials with results, Age and Gender are almost universally reported, however multiple reporting formats are used. Additional parameters are much less frequently reported. This may be owing to the use of “custom” measure definitions (e.g. “Fitzpatrick Skin Type”). The majority of these custom variables appear only once in the Clinical trials database. Indeed, we compare here a broad and heterogeneous collection of trials, which may have little in common, However, considerable variability in reporting pattern can be observed in published reports of trials around similar populations and research questions as also noted by [11–16].

Furthermore, our results show that ClinicalTrials.gov records and their corresponding journal articles differ substantially in the selection of reported measures and their format. On average, there are about twice as many measures included in journal articles than in the corresponding ClinicalTrials.gov record. Thus, we assert that ClinicalTrials.gov reports include only some of the parameters considered by researchers important enough to be published.

ClinicaTrials.gov is an invaluable resource for patients, their families and healthcare providers to navigate the complex and dynamic world of clinical trials. However, ClinicalTrials.gov targets researchers as well, providing a platform for conducting across-study analyses including meta-analyses, general [17] and domain-specific reviews. [18] To be generalizable, participant population reporting should reflect the population for investigated interventions [19], yet this is sometimes not the case. [20] as is also supported by our study. ClinicalTrials.gov can help researchers quantify the representation of populations of interest in trials. Tools to facilitate across-trial analysis have been designed.[21] Importantly, ClinicalTrials.gov is uniquely positioned to assess publication bias.[22] This is because unpublished studies are equally subject to mandatory reporting. Meta-analyses done on the ClinicalTrials.gov platform can thus potentially provide less biased results.

Yet, a pre-requisite for any across-trial analysis is that trial reports are comparable. As long as many different formats are used to report baseline measures, any attempt to pool this data from multiple trials would require manual alignment of variables. Thus, aligning the 178 different Age formats found in our work is labor intensive and error-prone. Furthermore, our results suggest that the discrepancies between format of baseline measures in published articles and ClinicalTrials.gov may hinder the use of ClinicalTirals.gov as an authoritative resource for secondary analyses of journal articles.

In this sense, the final rule is incomplete. While expanding mandatory reporting requirements, it does not mandate the use of uniformly coded, machine interpretable format in result reporting. Following the Notice of Proposed Rulemaking (NPRM) for Clinical Trials Registration and Results Submission, nearly 900 comments were received. The NPRM invited comments on the adequacy of the lists of proposed choices for Measure Type and Measure of Dispersion, however there were no specific comments on this topic. [3]

There is an inherent tension central to this matter. On one hand, secondary use of ClinicalTrials.gov requires standard formatting of trial reports. On the other, those conducting and sponsoring clinical trials want to have the freedom to collect, analyze and report their data as they see fit. These conflicting approaches are reflected in the comments made on the NPRM. [3] Authors of the final rule state that they generally agree with comments calling for maintaining flexibility in reporting results. This is in line with an objective of the final rule to improve the poor adherence rates to reporting of results on ClinicalTrials.gov. Admittedly, adopting stricter reporting requirements may have a negative effect on adherence, as it may require reporters to change the format of their results. However, reporters may arguably be more willing to invest and extra effort in this task if there is added value to it in the form of an increased capacity of ClinicalTrials.gov to support secondary use of trials.

Formulating the format of a structured results report is far from being straight forward. Considering the diversity of trials, aligning all components of their reports can also pose a real challenge. For example, when comparing trial-unique outcome definitions, such alignment may not even be possible. Yet when participant baseline measures are concerned, there can potentially be a core set defined for the most common and useful measures for machine-understandable, uniform reporting format. Experience from past shows, that when the National Library of Medicine (NLM) and the scientific community commit to the task, a standard nomenclature is generally definable. Successful terminology standardization including LOINC for lab results [23] and the Observational Medical Outcomes Partnership (OMOP) for medical records [24] are such example.

In the digital age, unlike in printed press, space is unlimited. The redundancy-tolerant digital medium can support a parallel, flexible reporting scheme. This means that researchers reporting trials results can use any format they deem right, as long as this is done in addition to-, and not instead of- the core uniform format.

Adherence to posting results of studies listed on ClinicalTrials.gov is poor, yet even among studies reporting results, inconsistent formats make automated across-trial analysis of these data impractical. The final rule aims to improve adherence to results reporting but does not solve the problem of multiple reporting formats. Our findings call for a discussion around implementing standards for unambiguous reporting of at least a core set of measures to support clinically and scientifically important secondary use of the ClinicalTrials.gov repository. Defining the set of measures to be reported and the format to be used should involve the entire research community, including funders and researchers.

## Supporting information

S1 Table Derivation of analysis population and summary of exclusion criteria. (DOCX)

S2 Table Formats of trial participant “Age” on ClinicalTrials.gov. (DOCX)

S3 Table Frequency of aggregated baseline characteristics reported in >1 study in the HLACTs subset of ClinicalTrials.gov studies (N = 13,808). (DOCX)

S4 Table Domain expert comparison between 30 ClinicalTrials.gov records and their corresponding journal articles. (DOCX)
