# Supplementary material for: Second thoughts on the final rule: An analysis of baseline participant characteristics reports on ClinicalTrials.gov
Source: PLoS One. 2017 Nov 6;12(11):e0185886. doi: 10.1371/journal.pone.0185886 (PMC5673198; doi:10.1371/journal.pone.0185886)
Supplement: S1 Table — (DOCX) [file pone.0185886.s001.docx]

**S1 Table: Derivation of Analysis Population and Summary of Exclusion Criteria**

| **Exclusion Criteria** | **No. remaining** |
| --- | --- |
| Studies downloaded from ClinicalTrials.gov | 160,145 (distinct) |
| Exclude: Overall recruitment status=WITHDRAWN | 157,133 |
| Exclude: Primary completion data <= 12/2007, or if missing , completion date <= 12/2007 | 116,676 |
| Exclude: Study type not INTERVENTIONAL | 92,064 |
| Exclude: Phase 0 or Phase 1 | 79,375 |
| Exclude: No US FDA oversight*, and (only non-US sites or no biological/device/drug/genetic/radiation intervention) | 24,238 |
| Exclude: Overall recruitment status not COMPLETED or TERMINATED | 13,855 |
| Exclude: Primary completion and completion dates missing, and verification date <= 12/2007 | 13,818 |
| Studies that are highly likely to be Applicable Clinical Trials (ACTs) and completed/terminated before 2016 | 13,818 |
