# Supplementary material for: Second thoughts on the final rule: An analysis of baseline participant characteristics reports on ClinicalTrials.gov
Source: PLoS One. 2017 Nov 6;12(11):e0185886. doi: 10.1371/journal.pone.0185886 (PMC5673198; doi:10.1371/journal.pone.0185886)
Supplement: S2 Table — (DOCX) [file pone.0185886.s002.docx]

**S2 Table: Formats of trial participant “Age” on ClinicalTrials.gov**

| BASELINE_MEASURE_TITLE | UNITS_OF_MEASURE | MEASURE_TYPE | DISPERSION |
| --- | --- | --- | --- |
| Age |  |  |  |
| Age | Age (years) | Mean | Standard Deviation |
| Age | age in years at baseline | Mean | Standard Deviation |
| Age | Days | Mean | Full Range |
| Age | Days | Mean | Standard Deviation |
| Age | days | Median | Full Range |
| Age | days | Median | Inter-Quartile Range |
| Age | days | Number |  |
| Age | days of gestation | Median | Full Range |
| Age | eyes | Number |  |
| Age | gestational age (weeks) | Median | Inter-Quartile Range |
| Age | gestational age in weeks | Mean | Standard Deviation |
| Age | hours | Mean | Standard Deviation |
| Age | month | Median | Full Range |
| Age | Month | Number |  |
| Age | Months | Mean | Full Range |
| Age | Months | Mean | Standard Deviation |
| Age | months | Median | Full Range |
| Age | months | Median | Inter-Quartile Range |
| Age | months | Median | Standard Deviation |
| Age | NA | Mean | Standard Deviation |
| Age | Number | Number |  |
| Age | number of participants | Number |  |
| Age | number of subjects | Number |  |
| Age | Participant | Number |  |
| Age | participants | Mean | Full Range |
| Age | participants | Mean | Standard Deviation |
| Age | participants | Median | Full Range |
| Age | Participants | Median | Inter-Quartile Range |
| Age | participants | Median | Standard Deviation |
| Age | participants | Number |  |
| Age | participants | Number |  |
| Age | patient participants | Number |  |
| Age | patients | Number |  |
| Age | subjects | Number |  |
| Age | weeks | Mean | Full Range |
| Age | weeks | Mean | Standard Deviation |
| Age | weeks | Median | Full Range |
| Age | weeks | Median | Inter-Quartile Range |
| Age | weeks | Number |  |
| Age | weeks (postnatal age) | Mean | Standard Deviation |
| Age | weeks gestation | Mean | Standard Deviation |
| Age | weeks of gestation | Mean | Standard Deviation |
| Age | Year | Mean | Full Range |
| Age | Year | Mean | Standard Deviation |
| Age | year | Median | Full Range |
| Age | year | Median | Inter-Quartile Range |
| Age | Year | Median | Standard Deviation |
| Age | years | Geometric Mean | Standard Deviation |
| Age | years | Mean | Full Range |
| Age | years | Mean | Inter-Quartile Range |
| Age | years | Mean | Standard Deviation |
| Age | years | Median | Full Range |
| Age | years | Median | Inter-Quartile Range |
| Age | years | Median | Standard Deviation |
| Age | Years | Number |  |
| Age | Years (yr) | Mean | Standard Deviation |
| Age | Years of Age | Median | Full Range |
| Age | years old | Mean | Standard Deviation |
| Age | years | Mean | Standard Deviation |
| Age (categorical) | participants | Number |  |
| Age (Statistics) | years | Mean | Standard Deviation |
| Age (years) (categorical) | participants | Number |  |
| Age - Part 1 population | years | Mean | Standard Deviation |
| Age 1st Drinking (years) | years | Mean | Standard Deviation |
| Age at Asthma Onset | years | Mean | Standard Deviation |
| Age at Beginning of Long term Follow-up Period | years | Mean | Standard Deviation |
| Age at chronic kidney disease diagnosis | years | Mean | Standard Deviation |
| Age at diagnosis | years | Median | Inter-Quartile Range |
| Age at Diagnosis of autosomal dominant polycystic kidney disease (ADPKD) | years | Mean | Standard Deviation |
| Age at Diagnosis of Autosomal Dominant Polycystic Kidney Disease (years) | years | Mean | Standard Deviation |
| Age at diagnosis of prostate cancer | years | Mean | Standard Deviation |
| Age at Enrollment | participants | Number |  |
| Age at Epilepsy Onset (years) | years | Mean | Standard Deviation |
| Age at First Diagnosis of Schizophrenia | years | Mean | Standard Deviation |
| Age at First Episode | years | Mean | Standard Deviation |
| Age at first major depressive disorder (MDD) episode | years | Mean | Inter-Quartile Range |
| Age at First Major Depressive Episode | years | Mean | Standard Deviation |
| Age at First Sexual Intercourse | Participants | Number |  |
| Age at HIV-1 Diagnosis | years | Mean | Standard Deviation |
| Age at index stroke | years | Mean | Standard Deviation |
| Age at Initial Egg Allergic Reaction | years | Mean | Standard Deviation |
| Age at Initial Peanut Allergic Reaction | years | Mean | Standard Deviation |
| Age at Onset | years | Mean | Standard Deviation |
| Age at Onset of ADHD | Participants | Number |  |
| Age at Onset of Attention-Deficit/Hyperactivity Disorder (ADHD) | years | Mean | Standard Deviation |
| Age at onset of Binge Eating Disorder (BED) | years | Mean | Standard Deviation |
| Age at onset of diabetes | years | Mean | Standard Deviation |
| Age at onset of hearing loss | years | Mean | Full Range |
| Age at Onset of Menopause | years | Mean | Standard Deviation |
| Age at Onset of Menopause by Category | participants | Number |  |
| Age at Onset of Restless Legs Syndrome | years | Mean | Standard Deviation |
| Age at onset, Bipolar I Disorder | years | Mean | Standard Deviation |
| Age at Primary Diagnosis | participants | Number |  |
| Age at radiotherapy | years | Median | Inter-Quartile Range |
| Age at receipt of Bacille Calmette-Guerin (BCG) vaccination (days) | participants | Number |  |
| Age at sexual debut | Years | Median | Full Range |
| Age at shunt palliation | days | Mean | Standard Deviation |
| Age at starting vasomotor symptoms (VMS), years of age | participants | Number |  |
| Age at surgery | years | Median | Inter-Quartile Range |
| Age at Time of Onset of First Episode | years | Mean | Standard Deviation |
| Age categorical | participants | Number |  |
| Age categories | participants | Number |  |
| Age categorization Females Only | participants | Number |  |
| Age Categorization, Female Only | Participants | Number |  |
| Age Characterization Female Only | participants | Number |  |
| Age Customized | participants | Number |  |
| Age Customized, by Disease Type | Participants | Number |  |
| Age first treated for depression | years | Mean | Standard Deviation |
| Age first treated for mania/hypomania | years | Mean | Standard Deviation |
| Age group | participants | Number |  |
| Age of depression onset | years | Mean | Standard Deviation |
| Age of Epilepsy Onset | years | Mean | Standard Deviation |
| Age of First Episode of MDD | years | Mean | Standard Deviation |
| Age of mania/hypomania onset | years | Mean | Standard Deviation |
| Age of onset | years | Mean | Standard Deviation |
| Age of onset of asthma | years | Mean | Standard Deviation |
| Age of Onset of Illness | years | Mean | Standard Deviation |
| Age of onset of regular drinking | years | Mean | Standard Deviation |
| Age of onset, mean age | Years | Mean | Standard Deviation |
| Age of participants | years | Mean | Standard Deviation |
| Age of participants by 10-year age groups | Participants | Number |  |
| Age of Smoking Onset | years | Mean | Standard Deviation |
| age of subjects with prior history of herpes labialis outbreak | participants | Number |  |
| Age of Type 1 Diabetes Diagnosis | years | Mean | Standard Deviation |
| Age of Vascular Access Graft | Years | Mean | Standard Deviation |
| Age of Wound | days | Median | Full Range |
| Age Strata | Participants | Number |  |
| Age, categorical: Period 2 (trial enrollment, induction ART) | participants | Number |  |
| Age, categorical: Period 3 (randomization to once vs twice daily ABC+3TC) | participants | Number |  |
| Age, categorical: Period 4 (randomization to stop versus continue cotrimoxazole) | participants | Number |  |
| Age, continuous: Period 2 (trial enrollment, induction ART) | years | Median | Inter-Quartile Range |
| Age, continuous: Period 3 (randomization to once vs twice daily ABC+3TC) | years | Median | Inter-Quartile Range |
| Age, continuous: Period 4 (randomization to stop versus continue cotrimoxazole) | years | Median | Inter-Quartile Range |
| Age, Customized |  |  |  |
| Age, Customized | Age at least 18 years | Number |  |
| Age, Customized | blood samples | Number |  |
| Age, Customized | children | Number |  |
| Age, Customized | Days | Mean | Full Range |
| Age, Customized | days | Mean | Standard Deviation |
| Age, Customized | days | Median | Full Range |
| Age, Customized | hour | Mean | Standard Deviation |
| Age, Customized | Hours | Mean | Standard Deviation |
| Age, Customized | Mean | Mean | Standard Deviation |
| Age, Customized | mean years of age | Mean | Standard Deviation |
| Age, Customized | months | Mean | Standard Deviation |
| Age, Customized | months | Number |  |
| Age, Customized | Number of Participants | Number |  |
| Age, Customized | Participants | Number |  |
| Age, Customized | Participants | Number |  |
| Age, Customized | Participant | Number |  |
| Age, Customized | participants | Mean | Standard Deviation |
| Age, Customized | participants | Median | Full Range |
| Age, Customized | participants | Median | Standard Deviation |
| Age, Customized | participants | Number |  |
| Age, Customized | participants with images collected | Number |  |
| Age, Customized | Participants | Number |  |
| Age, Customized | patients | Number |  |
| Age, Customized | percentage of participants | Number |  |
| Age, Customized | subjects | Number |  |
| Age, Customized | weeks | Mean | Full Range |
| Age, Customized | weeks | Mean | Standard Deviation |
| Age, Customized | weeks | Median | Full Range |
| Age, Customized | weeks' gestational age | Mean | Standard Deviation |
| Age, Customized | Year | Mean | Standard Deviation |
| Age, Customized | year | Median | Full Range |
| Age, Customized | years | Mean | Full Range |
| Age, Customized | years | Mean | Inter-Quartile Range |
| Age, Customized | years | Mean | Standard Deviation |
| Age, Customized | years | Median | Full Range |
| Age, Customized | years | Median | Inter-Quartile Range |
| Age, Customized | Years | Median | Standard Deviation |
| Age, Customized | Years | Number |  |
| Age, Customized | Years (Min, Max) | Median | Full Range |
| Age-adjusted Charlson Comorbidity Index | Points | Median | Inter-Quartile Range |
| Age-Categorical | participants | Number |  |
| AgeCategorical | participants | Number |  |
| AgeCategoricalOther | participants | Number |  |
