# Supplementary material for: Second thoughts on the final rule: An analysis of baseline participant characteristics reports on ClinicalTrials.gov
Source: PLoS One. 2017 Nov 6;12(11):e0185886. doi: 10.1371/journal.pone.0185886 (PMC5673198; doi:10.1371/journal.pone.0185886)
Supplement: S3 Table — (DOCX) [file pone.0185886.s003.docx]

**S3 Table: Frequency of aggregated baseline characteristics reported in >1 study in the HLACTs subset of ClinicalTrials.gov studies (N=13,808)**

| **Baseline measure type (aggregated)** | **No. (%) of studies reporting** |
| --- | --- |
| Age | 8545 (62) |
| Number of Participants | 6260 (45) |
| Gender | 6208 (45) |
| Region of Enrollment | 3881 (28) |
| Age, Customized | 723 (5) |
| Race/Ethnicity, Customized | 720 (5) |
| Race (NIH/OMB) | 611 (4) |
| Ethnicity (NIH/OMB) | 446 (3) |
| Body mass index | 266 (2) |
| Weight | 256 (2) |
| Height | 151 (1) |
| Performance Status | 113 (1) |
| Hemoglobin A1c | 72 (1) |
| Smoking Status | 64 (<1) |
| Gender, Customized | 53 (<1) |
| Duration of diabetes | 38 (<1) |
| Histology/Pathology | 38 (<1) |
| Systolic Blood Pressure | 21 (<1) |
| Menopausal status | 20 (<1) |
| Diastolic blood pressure | 17 (<1) |

HLACTs, Highly Likely Applicable Clinical Trials
