# Supplementary material for: Second thoughts on the final rule: An analysis of baseline participant characteristics reports on ClinicalTrials.gov
Source: PLoS One. 2017 Nov 6;12(11):e0185886. doi: 10.1371/journal.pone.0185886 (PMC5673198; doi:10.1371/journal.pone.0185886)
Supplement: S4 Table — (DOCX) [file pone.0185886.s004.docx]

**S4 Table: Domain expert comparison between 30 ClinicalTrials.gov records and their corresponding journal articles**

| **No.** | **Age report format match?** | | **Gender report format match?** | | **Total No.of baseline characteristics reported** | |
| --- | --- | --- | --- | --- | --- | --- |
|  | **y/n** | **Mismatch details** | **y/n** | **Mismatch details** | **ClinicalTrials.gov entry** | **Published paper** |
| 1 | y |  | n | F,M vs %F | 11 | 15 |
| 2 | y |  | n | F,M vs %F | 1 | 16 |
| 3 | y |  | n | F,M vs %M | 33 | 32 |
| 4 | y |  | n | F,M vs %F/%M | 7 | 15 |
| 5 | y |  | n | F,M vs %M | 4 | 11 |
| 6 | n | Mean, sd vs median, range | n | F,M vs Null | 2 | 11 |
| 7 | y |  | y |  | 4 | 5 |
| 8 | y |  | n | F,M vs Null | 4 | 16 |
| 9 | y |  | n | F,M vs %M | 5 | 13 |
| 10 | y |  | n | F,M vs %M | 0 | 19 |
| 11 | y |  | n | F,M vs F/%F | 5 | 11 |
| 12 | n | Mean, sd vs mean | n | F,M vs %M | 0 | 22 |
| 13 | y |  | y |  | 0 | 21 |
| 14 | y |  | n | F,M vs %M | 21 | 14 |
| 15 | y |  | n | F,M vs M/F | 0 | 19 |
| 16 | y |  | y |  | 1 | 10 |
| 17 | n | Null vs median, range | n | F,M vs Null | 7 | 23 |
| 18 | y |  | n | F,M vs %F | 18 | 12 |
| 19 | n | Mean, sd vs mean, range | y |  | 5 | 9 |
| 20 | y |  | n | F,M vs %M | 0 | 1 |
| 21 | n | Categories vs null | n | F,M vs Null | 13 | 8 |
| 22 | y |  | n | F/M vs %F | 0 | 7 |
| 23 | y |  | n | F,M vs %F | 4 | 8 |
| 24 | n | Mean, sd vs null | y |  | 1 | 9 |
| 25 | y |  | n | F,M vs %M | 12 | 9 |
| 26 | y |  | n | F,M vs %M | 16 | 22 |
| 27 | n | Null vs mean, sd | n | F,M vs %M | 13 | 12 |
| 28 | y |  | n | F,M vs %F | 0 | 28 |
| 29 | y |  | n | F,M vs %M | 0 | 4 |
| 30 | y |  | n | F,M vs %F | 4 | 6 |

sd, standard deviation; F, female; M, male
